# Utility of Point-of-Care Malaria Rapid Diagnostic Tests

**DOI:** 10.4269/ajtmh.2010.10-0114a

**Published:** 2010-07

Dear Sir:

In their short report on the utility of a point-of-care malaria rapid diagnostic test (RDT) in HIV-infected adults, Mills and others conclude that “the rapid diagnostic test accurately ruled “in or out” malaria.”^1^ However, at a positive predictive value of 70.6% and a lower confidence limit of 44.0%, it is difficult to state that the test accurately rules in malaria.

At the “rule out” side, there is a moderate sensitivity of 85.7%, again with a disappointing lower confidence limit of 57.2%, which is caused by the low number of malaria cases. The high negative predictive value is probably caused by low pre-test probability (or malaria prevalence) in the study population (14 of 246, 5.6%). Thus, the probability of not having malaria before the test was already 94.4%, and the diagnostic gain offered by the RDT is limited to 4.7%.

Malaria is a life-threatening disease that requires prompt and adequate treatment. Two of 14 cases were missed; the confidence limits indicate that it might have been six. Rapid diagnostic tests can miss not only low parasite densities such as in the present study,^2^ but also high parasite densities caused by the prozone effect.^3^

A cautious conclusion of this study might be that RDTs might be potentially useful to rule out malaria, but that studies on larger samples and patient populations with higher malaria prevalence are necessary to confirm this finding.

J. Van den Ende

Jan Jacobs

Institute of Tropical Medicine

Antwerp, Belgium

E-mail: jvdende@itg.be

Zeno Bisoffi

Center for Tropical Diseases

Sacro Cuore Hospital

Negrar, Verona, Italy
